# The Injection of Lipids Into Yolk Sac has Been Shown to Alter the Lipid Metabolism of Adult Nile Tilapia (*Oreochromis niloticus*)

**DOI:** 10.1155/anu/8360989

**Published:** 2026-02-25

**Authors:** Linli Luo, Sirijanya Thongchaitriwat, Suksan Kumkhong, Janethida Kiatmontri, Shenglin Yang, Stephane Panserat, Surintorn Boonanuntanasarn

**Affiliations:** ^1^ School of Animal Technology and Innovation, Institute of Agricultural Technology, Suranaree University of Technology, Muang, Nakhon Ratchasima, 30000, Thailand, sut.ac.th; ^2^ Key Laboratory of Animal Genetics, Breeding and Reproduction in the Plateau Mountainous Region, Ministry of Education, Guizhou University, Guiyang, 550025, China, gzu.edu.cn; ^3^ National Research Institute for Agriculture Food and Environment, Université de Pau and Pays de L’Adour, NuMeA Aquapôle, Saint-Pée-sur-Nivelle, 64310, France

**Keywords:** fish oil, linseed oil, lipid metabolism, microinjection, *n*−3 PUFA, nutritional programming

## Abstract

Nutritional programming (NP) of *n*−3 polyunsaturated fatty acids (PUFAs), achieved by injecting linseed oil into Nile tilapia alevins, influences lipid profiles and associated metabolic processes during the juvenile stage. However, the persistence of these effects into adulthood is unknown. In this study, we investigated the long‐term NP effects of early *n*−3 PUFA and *n*−3 long‐chain (LC)‐PUFA intervention via linseed and fish oil injection, respectively, during the alevin stage on lipid metabolism and associated pathways in adult Nile tilapia. The experimental design included randomized treatment groups of 0.85% NaCl (control), linseed oil, and fish oil, each with six replicates. Linseed and fish oil were microinjected into the yolk reserves of Nile tilapia alevins, while control fish received NaCl injections. Following dietary challenge with a linseed oil‐rich diet (weeks 37–40), linseed oil‐injected fish exhibited higher weight gain, suggesting that early linseed oil enrichment enhanced *n*−3 PUFA utilization for growth. Both interventions reduced plasma lipemia, promoted hepatic fat accumulation, and downregulated *mlxipl* and *acaca* expression in the muscle, potentially modulating interactions between carbohydrate and lipid metabolism. While these effects were more pronounced in the fish oil‐injected group, long‐term NP effects differed between the liver and muscle, including decreased hepatic but increased muscular *n*−3 LC‐PUFA deposition, and downregulated hepatic but upregulated muscular β‐oxidation in fish oil‐injected adult fish. Gene expression analysis revealed altered hepatic enzymes involved in DNA (de)methylation and histone modification, implicating epigenetic mechanisms in the long‐term NP effects of early *n*−3 PUFA and *n*−3 LC‐PUFA exposure. Thus, linseed and fish oil enrichment during the alevin stage induces long‐term alterations in lipid metabolism and enhances muscular *n*−3 LC‐PUFA deposition in adult Nile tilapia.

## 1. Introduction

Fish, particularly marine species, are high‐quality sources of protein and are enriched in *n*−3 long‐chain polyunsaturated fatty acids (LC‐PUFAs), which are beneficial for human health [1]. For sustainable aquaculture, and considering the limitations of capture fisheries, farm‐raised fish can serve as an alternative source of *n*−3 LC‐PUFAs. However, intensive investigation of lipid metabolism is required to determine the qualitative and quantitative requirements for essential fatty acids (EFAs) in farmed fish for optimal fatty acid profiles and to maximize growth and health benefits [2, 3]. Further, it is challenging to produce farmed fish as a sustainable source of *n*−3 LC‐PUFAs—particularly eicosapentaenoic acid (EPA; C20:5*n*−3) and docosahexaenoic acid (DHA; C22:6*n*−3)—as a substitute for wild‐fish‐derived sources [4]. In addition, investigating the de novo biosynthesis of dietary linolenic acid (C18:3*n*−3) and linoleic acid (C18:2*n*−6) into their respective LC forms (*n*−3 LC‐PUFA and *n*−6 LC‐PUFA) may serve as a potential source of LC‐PUFAs for both aquafeeds and human nutrition [5, 6]. Furthermore, strategies aimed at modulating lipid metabolism to enable efficient utilization of alternative dietary lipids—thus enhancing LC‐PUFA biosynthesis and tissue deposition—have become a growing area of interest in aquaculture to ensure global LC‐PUFA supply.

Nutritional programming (NP) refers to the long‐term impact of nutritional or environmental stimuli during critical developmental windows on growth, development, and metabolism later in life [7] and exerts long‐term effects on metabolism in animals, including fish [8]. The regulation of lipid metabolism through NP has been demonstrated in marine fish. For example, feeding gilthead sea bream (*Sparus aurata*) a linseed oil‐rich diet alters egg fatty acid composition, increases hepatic LC‐PUFA content and modifies lipid metabolism in both 4‐month‐old and 16‐month‐old fish close to their first breeding season [9–11]. Parental NP in gilthead sea bream has been studied by replacing fish oil with linseed oil in broodstock diets. NP modulates several aspects of lipid metabolism, including hepatic lipoprotein lipase downregulation, very LC fatty acids (*elovl*) elongation, fatty acid desaturase 2 (*fads2*) expression, and improved utilization of diets deficient in fishmeal and fish oil [12]. In large yellow croaker (*Larimichthys crocea*), feeding larvae with a mixture of soybean oil and linseed oil significantly increases larval *n*−3 PUFA content. Moreover, a subsequent dietary challenge modulates the hepatic expression of lipid‐metabolism‐related genes and increases *n*−3 PUFA deposition in both the liver and muscle relative to a fish oil‐fed control group [13]. While NP effects on lipid metabolism have been extensively studied in marine fish, their effects in freshwater species remain incompletely understood. Marine fish possess a higher total content of *n*−3 PUFA than that of *n*−6 PUFA; in contrast, freshwater fish exhibit lower total *n*−3 PUFA content than that of *n*−6 PUFA [14]. Globally, capture fisheries are largely marine‐based; however, inland aquaculture production has surpassed that of marine aquaculture [15]. Therefore, exploring NP strategies to modulate lipid metabolism and fatty acid profiles in freshwater fish may offer promising avenues to support the sustainable supply of *n*−3 PUFAs for global consumption.

Among global freshwater aquaculture species, tilapia is the second most widely farmed fish, with Nile tilapia (*Oreochromis niloticus*) dominating the overall tilapia production. Nile tilapia farming faces challenges in terms of increasing yields as well as enhancing product quality, particularly fatty acid composition. Although Nile tilapia contains approximately 2% fat in the flesh, lipid metabolism for increasing LC‐PUFA content in this species has been intensively studied. For example, the dietary EFA requirements for Nile tilapia are 0.45%–0.64% for α‐linolenic acid (C18:3*n*−3) and 0.5% for linoleic acid (C18:2*n*−6) or arachidonic acid (C20:4*n*−6) [16–18]. Replacing fish oil with linseed oil in up to 50% of the diet improves growth and increases muscle n‐3 PUFA, particularly C18 : 3n–3, but not EPA or DHA [19]. While various dietary lipid sources such as soybean, linseed, and palm oil influence hepatic fatty acid composition, no significant effects on adult growth have been observed. However, proteomic analyses of the liver and plasma indicated that dietary lipid sources modulate physiological functions related to oxidative stress, immune response, and inflammation [20, 21]. Functional characterization of *fads2* in Nile tilapia revealed that its recombinant yeast expression exhibits *Δ*6 and *Δ*5 desaturase activities toward C18:2*n*−6 and C18:3*n*−3, respectively [6, 22]. Thus, further elucidation of lipid metabolism—particularly its modulation—is required, with NP being a promising approach. Early glucose injection during the alevin stage exerts strong long‐term NP effects on carbohydrate intermediary metabolism in both juvenile and adult stages [23, 24].

As fish are an important source of *n*−3 LC‐PUFAs, understanding the metabolic responses that enhance *n*−3 LC‐PUFA levels in fish is of considerable interest. It is important to investigate how nutritional interventions in freshwater species affect metabolic responses and fatty acid profiles. In a previous study, injection of linseed oil, an *n*−3 PUFA source, into Nile tilapia alevins exerted long‐term effects on lipid metabolism in juvenile fish, significantly increasing the hepatic levels of *n*−3 PUFA, particularly C18:3*n*−3, EPA, and DHA [25]. In this study, we investigated the longevity of the stimulatory effects of linseed oil. To explore how NP modulates lipid metabolism related to LC‐PUFA biosynthesis, we evaluated the response of adult fish to a linseed oil‐rich diet containing substrates for LC‐PUFA biosynthesis. In addition, we elucidated the long‐term effects of NP via fish oil (*n*−3 LC‐PUFA source) injection into yolk reserves during the alevin stage on lipid metabolism in adult Nile tilapia. These findings demonstrate comparative long‐term effects of early linseed and fish oil exposure on adult growth, intermediary and lipid metabolism, and fatty acid deposition in the liver and muscle. At the molecular level, our results indicate that NP influences the expression of enzymes involved in DNA (de)methylation and histone modification, suggesting potential epigenetic regulation in mediating the long‐term effects of early lipid exposure.

## 2. Materials and Methods

### 2.1. Ethics Statement and Experimental Study Design

All experimental protocols were approved by the Ethics Committee of Suranaree University of Technology, Animal Care and Use Committee (Approval Number A‐18/2562). Nile tilapia (*Oreochromis niloticus*) broodstock (body weight ~0.8–1.2 kg) were maintained in an 800 m^2^ earthen pond at the Suranaree University of Technology Farm, Nakhon Ratchasima, Thailand. During the breeding period, brood stock were fed a commercial diet containing 30% crude protein (CP) and 4% crude fat (CF; Table 1) once daily.

**Table 1:** Fatty acid composition of injected oils and ingredients and proximate composition of challenging diets in this study.

Ingredients (%)	Linseed oil	Fish oil	Challenging diet (linseed oil‐rich diet)
Fish meal	—	—	30.0
Soybean meal	—	—	26.0
Rice flour	—	—	30.0
Rice bran	—	—	12.0
Linseed oil	—	—	3.0
Fish premix^a^	—	—	1.0
Vitamin C	—	—	1.0
Proximate composition (%)			
Dry matter	—	—	97.5
Protein	—	—	32.2
Fat	—	—	8.5
Fiber	—	—	4.1
Ash	—	—	11.2
NFE^b^	—	—	41.5
Gross energy (kJ g^−1^)	—	—	16.8
Fatty acids (mg/100 g lipids)			
C14:0	—	2.62	2.49
C16:0	3.13	6.79	6.15
C16:1	0.14	3.44	0.73
C18:0	2.33	1.46	2.72
C18:1*n*−9	12.08	4.64	8.88
C18:2*n*−6	11.59	0.74	7.11
C18:3*n*−3	31.48	0.14	12.23
C18:3*n*−6	0.45	0.20	0.08
C18:4*n*−3	—	0.95	0.02
C20:1*n*−9	—	0.15	0.18
C20:3*n*−6	0.24	—	0.03
C20:3*n*−3	—	—	0.03
C20:4*n*−6	0.19	0.52	0.48
C20:5*n*−3	—	5.50	0.22
C22:6*n*−3	—	5.21	1.40
SFA	5.46	10.87	11.36
MUFA	12.22	8.23	9.79
* n*−3 PUFA	31.48	11.80	13.90
* n*−6 PUFA	12.47	1.46	7.70

Abbreviations: MUFA, monounsaturated fatty acid; PUFA, polyunsaturated fatty acid; SFA, saturated fatty acid.

^a^Vitamin and trace mineral mix included the following (IU kg^−1^ or g kg^−1^ diet): biotin, 0.25 g; folic acid, 0.003 g; inositol, 0.25 mg; niacin, 0.0215 g; pantothenic acid, 0.03 g; vitamin A, 5000 IU; vitamin B1, 0.0025 g; vitamin B2, 0.0012 g; vitamin B6, 0.0075 g; vitamin B12, 0.00005 mg; vitamin C, 1 g; vitamin D3,1000 IU; vitamin E, 100 IU; vitamin K, 0.008 g; copper, 0.02 g; iron, 0.2 g; selenium, 0.3 mg; zinc, 0.32 g.

^b^Nitrogen‐free extract = dry matter − (crude protein + crude lipid + crude fiber + ash).

To investigate metabolic responses to *n*−3 PUFA and *n*−3 LC‐PUFA, we used a randomized experimental design with three treatment groups: 0.85% NaCl (control), linseed oil, and fish oil, each with six replicates (Figure 1A). Fertilized eggs were obtained from female Nile tilapia (used as biological replicates) and incubated in hatching trays (20 × 30 × 5 cm^3^) with flow‐through freshwater maintained at 27–29°C. Alevins at developmental stage 17, as described by Fujimura and Okada [26], were used for microinjection. Sixty nanoliters of 0.85% NaCl, linseed oil, or fish oil (Table 1) was microinjected into the yolk sac (100 larvae per replicate) using a micromanipulator (MP‐2R) and microinjector (IM‐9A; Narishige, Tokyo, Japan; Figure 1B) following the procedures of Kumkhong et al. [23, 24]. After microinjection, both microinjected and noninjected alevins were reared in hatching trays for 1 week postinjection (1 wpi). Subsequently, the experimental fry at 2 wpi were gently transferred to individual cages (40 × 40 × 60 cm^3^), serving as replicates. These cages were placed in cement ponds (2 × 2 × 0.8 m^3^) with continuous aeration. To eliminate confounding effects due to sexual dimorphism in Nile tilapia, all fry were subjected to masculinization during 2–5 wpi using a commercial powdered diet (Table 1; 40% CP, 8% CF) mixed with 17α‐methyltestosterone at 60 mg kg^−1^. Feed was administered five times daily (09:00, 11:00, 13:00, 15:00, and 17:00) to ensure uniform growth and sex reversal to produce all‐male fish [27]. After sex reversal (through 5 wpi), survival rate of all experimental fry was recorded to determine whether early microinjection of linseed oil or fish oil had detrimental effects. Survival rates of microinjected alevins were significantly lower than those of uninjected alevins (*p* < 0.05). However, no significant differences were observed in survival among the microinjected treatment groups (Supporting Information 1: Figure S1). Thus, yolk injection with LC‐PUFA‐rich oils did not induce additional mortality. Consequently, the experimental fry were reared following standard practices in Nile tilapia aquaculture.

Figure 1Experimental design illustrating the early‐life injection (nutritional programming history) of linseed oil or fish oil and the subsequent dietary linseed oil‐rich challenge (A). Newly hatched Nile tilapia alevins were injected with either saline (0.85% NaCl), linseed oil, or fish oil into the yolk reserve (B). The fish were reared and fed the following commercial diets: 40% crude protein (CP) and 8% crude fat (CF) from 2 to 8 weeks postinjection (wpi) and 32% CP and 4% CF from 9 to 36 wpi through to adulthood. All experimental fry were fed a diet supplemented with 60 mg kg^−1^ of 17α‐methyltestosterone during weeks postinjection (wpi) 2–5. The survival rate of the experimental fry was determined during week postinjection (wpi) 5 (Supporting Information 3: Figure S1). During 37–40 wpi, a dietary challenge was performed using a linseed oil‐rich diet (Table 1). Fish were sampled at 40 wpi (after the challenge) for blood chemistry, proximate composition, fatty acid profiling, and gene expression analysis related to fatty acid metabolism and epigenetic modification. CF,crude fat; CP,crude protein; wpi, weeks postinjection.

**Figure:**
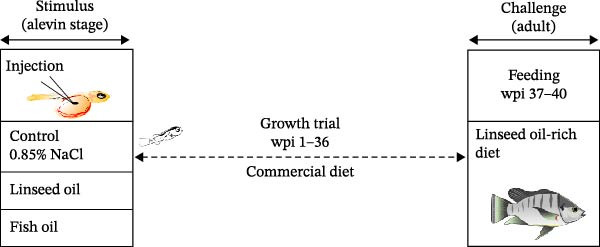
(A)

**Figure:**
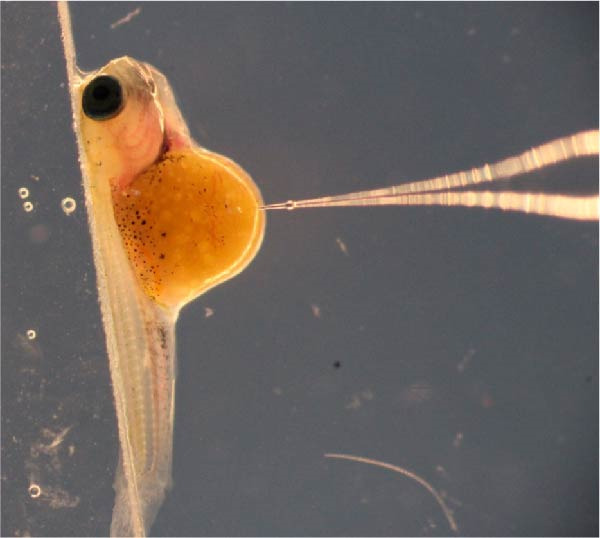
(B)

### 2.2. Fish Culture and Experimental Diet

From 6–8 wpi, fry continued to receive the hormone‐free powdered diet. From 9 to 36 wpi, fish from each cage (replicate) were reared in cement ponds (2 × 2 × 0.8 m^3^) and fed a commercial diet (Table 1; 32% CP, 4% CF) ad libitum twice daily (09:00 and 16:00). Body weight was recorded every 4 weeks throughout the experimental period. During fish culture, air and water temperatures were measured daily, ranging from 16.4–37.0 and 18.0–29.3°C, respectively. Notably, the low water temperatures occurred only for a few hours at night and had no adverse effects on feeding behavior. A pH meter and dissolved oxygen (DO) meter were used to record pH and DO levels weekly; pH ranged from 7.1 to 8.3 and DO from 4.2 to 7.6 mg L^−1^.

A linseed oil‐rich diet (Table 1) was used for the challenge test during the adult stage. The formulation, proximate composition, and fatty acid profile of the diet are presented in Table 1. To assess the response of adult fish (37–40 wpi) to the challenge diet, 10 fish per replicate (*n* = 10/replicate; six replicates) were randomly selected and cultured in individual cages (90 × 80 × 110 cm^3^). Fish were fed the linseed oil‐rich diet, and growth was measured before and after the feeding trial. During the challenge period, air and water temperatures were recorded daily, ranging from 30.0–34.0 and 27.5–29.0°C, respectively. pH and DO were measured weekly; pH ranged from 7.2 to 7.9 and DO from 4.2 to 4.5 mg L^−1^.

### 2.3. Fish Sampling, Blood Collection, and Proximate Composition

To analyze the fatty acid composition, alevins at 1 wpi were randomly sampled (three whole‐body alevins pooled per replicate), immediately frozen in liquid nitrogen, and stored at −80°C until analysis. As the 5‐h postprandial period corresponded to the peak in postprandial glycemia, fish sampling was conducted 5 h after feeding to assess metabolic responses related to feed and feeding [27]. At 36 wpi, after 5 h of feeding, three fish per replicate were euthanized for sampling. Of these, two fish per replicate were used for blood collection to analyze plasma metabolites. Blood samples were drawn from the caudal vein using syringes preloaded with K_2_EDTA anticoagulant (1.5 mg mL^−1^) and kept on ice. Plasma was separated by centrifugation at 9000 × *g* for 10 min at 4°C and stored at −80°C until analysis. Following blood collection, livers were excised and weighed to calculate the hepatosomatic index (HSI), then immediately frozen in liquid nitrogen and stored at −80°C for analysis of protein, fat, and ash content according to Association of Official Analytical Chemists (AOAC) [28]. Muscle samples were collected from two fish per replicate and stored at −80°C for proximate composition analysis. In addition, one fish per replicate was randomly selected and sampled for whole‐body proximate composition, following AOAC [28] procedures.

At 40 wpi, 5 h after feeding the linseed oil‐rich diet, five fish per replicate were euthanized. Of these, two fish per replicate were used for plasma metabolite analysis. Blood was collected from the caudal vein, and the liver was excised and weighed to calculate HSI, and then immediately frozen in liquid nitrogen and stored at −80°C. Liver and muscle were collected for nutrient composition analysis [28], fatty acid profiling, and RNA extraction. In addition, one fish per replicate was sampled for whole‐body proximate composition according to AOAC [28].

### 2.4. Blood Chemistry Analysis

We analyzed plasma metabolites (two fish per replicate) including glucose, triglycerides, cholesterol, total protein, blood urea nitrogen (BUN), serum aspartate aminotransferase (SGOT), and serum alanine aminotransferase (SGPT). Plasma glucose was determined using the glucose oxidase–peroxidase method [29]. Triglycerides were measured using the glycerol‐3‐phosphate oxidase–sodium N‐ethyl‐N‐(3‐sulfopropyl) m‐toluidine method [30]. Cholesterol was assessed using the cholesterol oxidase–phenol and aminophenazone method [31]. Total protein was analyzed using the biuret method [32]. BUN was determined using a modified indophenol colorimetric method [33]. SGOT and SGPT activities were estimated using colorimetric methods as described by Reitman and Frankel [34].

### 2.5. Fatty Acid Composition Analysis

Fatty acid composition was analyzed in whole‐body alevins at 1 wpi, and in liver and muscle of adult fish at 40 wpi, following Tanomman et al. [6]. In brief, samples were homogenized in chloroform:methanol (2:1, *v*/*v*) containing 0.01% butylated hydroxytoluene as an antioxidant. Extracted lipids were saponified and methylated by adding 100 mg lipid extract to 1.5 mL methanol containing 0.5 N NaOH at 100°C for 7 min, followed by esterification using 14% boron trifluoride (BF_3_) in methanol at 100°C for 5 min. Fatty acid methyl esters (FAMEs) were analyzed using a gas chromatograph (GC‐2014, Shimadzu, Japan) with a flame ionization detector and RESTEK Rtx‐Wax fused‐silica capillary column (30 m × 0.25 mm i.d. × 0.25 µm film; Restek Corporation, Bellefonte, PA, USA). Hydrogen carrier gas linear velocity and split ratio were 20.0 cm s^−1^ and 25:1, respectively. Injector and detector temperatures were 230 and 250°C. The column program was 140°C initial, ramp to 220°C at 4°C min^−1^, hold 220°C for 40 min. FAMEs were identified and quantified by comparison of their retention times versus those of FAME mix standards (Supelco Component FAME mix; Cayman Chemical Company, MI, USA). The fatty acid composition was calculated based on the area of each peak, and the amount was determined by comparison with the methyl heptadecanoate internal standard.

### 2.6. Total RNA Extraction and Quantitative Real‐Time Reverse Transcription

Liver (50 mg) and muscle (100 mg) from adult fish (two fish per replicate; *n* = 12 per group) were used for total RNA extraction using TRIzol (Invitrogen, Carlsbad, CA, USA). RNA concentration and purity were measured using NanoDrop spectrophotometer (Thermo Fisher Scientific, Madison, WI, USA) and verified on a 1% agarose gel. One µg RNA was reverse‐transcribed into complementary DNA (cDNA) using SuperScript III RNase H^−^ Reverse Transcriptase Kit (Invitrogen) with random primers (Promega, Charbonnières, France). Each RNA sample was reverse‐transcribed in duplicate (two cDNA replicates per RNA). Gene expression was quantified by real‐time reverse transcription PCR (qRT‐PCR) in duplicate (two qPCR replicates per cDNA) via the Roche E‐Method, based on Pfaffl [35]. Primer sequences are listed in Supporting Information 2: Table S1 and Supporting Information 3: Table S2. The reference gene *ef1*α did not differ among groups and was used for normalization. PCR efficiency (from standard‐curve slope) ranged 1.8–2.0.

Supporting Information 2: Table S1 lists primers for lipid metabolism and related pathways: (i) β‐oxidation: *cpt1cb*, *acox1*, and *pparα*; (ii) biosynthesis: *fads2*, *elovl6*, and *elovl7*; (iii) lipid transport: *mttp*; (iv) cholesterol synthesis: *hmgcs1*; (v) glucose–lipid interaction: *mlxipl* and *acaca*; (vi) eicosanoid synthesis: *alox5*.

Supporting Information 3: Table S2 lists primers for DNA methylation and histone modification: (i) DNA methyltransferases: *dnmt3aa* and *dnmt3bb*; (ii) TET dioxygenases: *tet1*, *tet2*, and *tet3*; (iii) H3K4me3 methyltransferases: *kmt2ba*, *kmt2bb*, *setd1a*, and *setd1ba*; (iv) H3K4me3 demethylases: *kdm5bb*, *kdm5c*, and *riox1*; (v) H3K9ac acetylases: *kat2b*, *kat6a*, and *gtf3c4*; (vi) H3K9ac deacetylases: *sirt2* and *sirt5*.

### 2.7. Statistical Analyses

Data were analyzed using SPSS for Windows, version 22.0 (SPSS Inc., Chicago, IL, USA). One‐way ANOVA assessed differences among treatment groups; when significant, Tukey’s HSD post hoc test ranked means. Statistical significance was set at *p* < 0.05. In addition, principal component analysis (PCA) of fatty acid profiles was performed using PCA Maker **(**Sumire Production LLC, Tokyo, Japan).

## 3. Results

### 3.1. Effects of Linseed Oil and Fish Oil Injection in Alevins on Fatty Acid Composition and Growth in Adult Fish

All experimental alevins were collected at 1 wpi for fatty acid analysis (Table 2). The fatty acid compositions of the uninjected control and 0.85% NaCl groups were similar. However, injection of linseed oil or fish oil into the yolk reserve altered fry fatty acid profiles at 1 wpi. Both injections increased saturated fatty acids (SFAs), monounsaturated fatty acids (MUFAs), *n*−3 PUFAs, *n*−6 PUFAs (only linseed oil), and the *n*−3/*n*−6 PUFA ratio (*p* < 0.05; Table 2). Compared with linseed oil, fish oil led to a greater increase in SFA (C14:0, C16:0), MUFA (C16:1), EPA, and DHA. Linseed oil increased C20:0, C18:2*n*−6, and C18:3*n*−3, relative to the fish oil group (*p* < 0.05).

**Table 2:** Fatty acid composition of alevin injected with saline (0.85% NaCl), linseed oil, and fish oil at 1 wpi (mean ± SD, *n* = 6).

Fatty acid (mg/100 g lipids)	Control un‐injection	0.85 % NaCl history	Linseed oil history	Fish oil history	*p*‐Value
C10:0	1.91 ± 0.27	1.99 ± 0.19	2.37 ± 0.11	2.52 ± 0.41	0.212
C12:0	0.92 ± 0.01^b^	0.93 ± 0.03^b^	1.16 ± 0.03^a^	1.12 ± 0.01^a^	**0.001**
C14:0	2.43 ± 0.21^c^	2.46 ± 0.18^c^	5.63 ± 0.39^b^	17.49 ± 0.07^a^	**<0.001**
C14:1	1.55 ± 0.10	1.55 ± 0.05	2.13 ± 0.36	2.30 ± 0.22	0.052
C16:0	65.36 ± 2.19^c^	65.99 ± 1.46^c^	84.26 ± 0.41^b^	193.43 ± 2.71^a^	**<0.001**
C16:1	5.85 ± 0.26^c^	5.79 ± 0.12^c^	8.50 ± 0.01^b^	10.63 ± 0.10^a^	**<0.001**
C18:0	23.63 ± 3.03	23.80 ± 2.89	31.53 ± 0.88	27.14 ± 3.45	0.122
C18:1*n*−9	44.84 ± 0.71^b^	44.19 ± 0.80^b^	53.77 ± 2.27^a^	50.01 ± 0.78^a^	**0.005**
C18:2*n*−6	26.02 ± 1.09^a,b^	25.61 ± 0.84^a,b^	28.51 ± 0.68^a^	24.94 ± 0.73^b^	**0.048**
C20:0	0.86 ± 0.02^a,b^	0.85 ± 0.03^b^	0.99 ± 0.05^a^	0.73 ± 0.00^b^	**0.008**
C18:3*n*−6	2.68 ± 0.01^b^	2.66 ± 0.02^b^	3.10 ± 0.09^a^	3.05 ± 0.10^a^	**0.006**
C20:1*n*−9	2.30 ± 0.03^c^	2.32 ± 0.02^c^	3.32 ± 0.10^b^	4.54 ± 0.04^a^	**<0.001**
C18:3*n*−3	27.74 ± 0.41^b^	28.21 ± 0.26^b^	95.78 ± 1.76^a^	31.03 ± 0.01^b^	**<0.001**
C18:4*n*−3	16.98 ± 3.67^b^	16.97 ± 3.24^b^	28.55 ± 0.84^a^	27.80 ± 0.25^a^	**0.015**
C20:2	58.10 ± 5.76^b^	63.41 ± 2.26^b^	90.33 ± 6.17^a^	75.20 ± 2.58^a,b^	**0.007**
C22:0	8.50 ± 0.37^b^	8.54 ± 0.15^b^	10.30 ± 0.31^a^	11.11 ± 0.19^a^	**0.001**
C20:3*n*−6	31.25 ± 1.93^b^	31.90 ± 0.68^b^	43.50 ± 2.03^a^	32.22 ± 1.47^b^	**0.004**
C22:1*n*−9	29.85 ± 0.79^b^	29.65 ± 0.66^b^	30.88 ± 0.88^b^	48.40 ± 1.01^a^	**<0.001**
C20:3*n*−3	23.53 ± 1.36	24.17 ± 0.35	26.59 ± 2.00	25.23 ± 0.40	0.216
C20:4*n*−6	2.04 ± 0.08^b^	2.16 ± 0.09^a,b^	2.30 ± 0.07^a,b^	2.33 ± 0.03^a^	**0.038**
C22:2	0.10 ± 0.00^b^	0.10 ± 0.00^b^	0.21 ± 0.01^a^	0.19 ± 0.01^a^	**0.001**
C20:5*n*−3	3.99 ± 0.06^c^	3.99 ± 0.11^c^	6.34 ± 0.56^b^	26.16 ± 1.4^a^	**<0.001**
C24:0	0.17 ± 0.05	0.16 ± 0.03	0.21 ± 0.02	0.21 ± 0.00	0.416
C24:1	0.51 ± 0.20^b^	0.52 ± 0.17^b^	1.08 ± 0.01^a^	1.48 ± 0.12^a^	**0.006**
C22:6*n*−3	3.01 ± 0.01^b^	3.05 ± 0.02^b^	5.57 ± 0.15^b^	25.80 ± 1.43^a^	**<0.001**
SFA	112.27 ± 3.92^c^	113.39 ± 0.98^c^	144.82 ± 0.59^b^	261.85 ± 6.9^a^	**<0.001**
MUFA	84.9 ± 0.09^c^	84.02 ± 0.01^c^	99.68 ± 2.89^b^	117.36 ± 0.19^a^	**<0.001**
PUFA	195.44 ± 4.83^c^	202.22 ± 6.37^c^	330.79 ± 1.84^a^	273.96 ± 3.93^b^	**<0.001**
*n*−3 PUFA	75.24 ± 1.83^c^	76.38 ± 2.49^c^	162.84 ± 1.48^a^	136.03 ± 3.47^b^	**<0.001**
*n*−6 PUFA	61.99 ± 0.91^b^	62.33 ± 1.62^b^	77.41 ± 2.87^a^	62.54 ± 2.12^b^	**0.004**
*n*−3/*n*−6 PUFA	1.21 ± 0.05^b^	1.23 ± 0.01^b^	2.10 ± 0.06^a^	2.18 ± 0.13^a^	**<0.001**
*n*−6/*n*−3 PUFA	0.82 ± 0.03^a^	0.82 ± 0.01^a^	0.48 ± 0.01^b^	0.46 ± 0.03^b^	**<0.001**

*Note:* Different superscript letters and bold values indicate significant differences in the mean values among experimental groups (*p* < 0.05).

Abbreviations: MUFA, monounsaturated fatty acid; PUFA, polyunsaturated fatty acid; SFA, saturated fatty acid; wpi, week postinjection.

Subsequently, fry injected with 0.85% NaCl, linseed oil, or fish oil were cultured and fed a commercial diet during 2–36 wpi. Growth was similar among groups during 4–36 wpi (28–252 days; data not shown). In adult fish fed a linseed oil‐rich challenge diet during 37–40 wpi, both linseed and fish oil treatments tended to improve final body weight, weight gain, and average daily gain (ADG), versus the 0.85% NaCl group, with significant differences observed only in the linseed oil group (*p* < 0.05; Table 3), although specific growth rate (SGR) and feed conversion ratio were similar. Survival did not differ among groups (Table 3).

**Table 3:** Growth performance and survival rate during 4–40 wpi among adult Nile tilapia injected with saline (0.85% NaCl), linseed oil, or fish oil during the alevin stage (mean ± standard deviation (SD), *n* = 6).

	0.85% NaCl history	Linseed oil history	Fish oil history	*p*‐Value
Initial weight (g)^1^	1.30 ± 0.14	1.36 ± 0.03	1.28 ± 0.05	0.248
Final weight (g)	387.92 ± 5.09^a^	399.68 ± 6.20^b^	393.68 ± 6.78^a,b^	**0.015**
Weight gain (g)^2^	386.63 ± 5.13^a^	398.40 ± 6.22^b^	392.32 ± 6.76^a,b^	**0.015**
ADG (g day^−1^)^3^	1.48 ± 0.02^a^	1.53 ± 0.02^b^	1.50 ± 0.03^a,b^	**0.012**
SGR (% day^−1^)^4^	2.19 ± 0.04	2.20 ± 0.02	2.17 ± 0.01	0.210
FI (g day^−1^)	2.05 ± 0.05	2.13 ± 0.05	2.10 ± 0.06	0.052
FCR^5^	1.39 ± 0.04	1.40 ± 0.03	1.40 ± 0.05	0.850
Survival rate (%)	88.89 ± 8.61	88.89 ± 8.61	94.44 ± 8.61	0.454

*Note:* Different superscript letters and bold values indicate significant differences in the mean values among experimental groups (*p*  < 0.05).

Abbreviations: FI , feed intake; wpi, week postinjection.

^1^Body weight was recorded as initial weight for growth performance at 4 wpi. During 1–4 wpi, the experimental larvae were fed with a 17‐MT supplemented diet to produce all male population, to prevent confounding effects from sex dimorphism for growth in adult fish.

^2^Weight gain = Final body weight − initial body weight.

^3^Average daily gain (ADG) = (Final body weight − initial body weight)/experimental days.

^4^Specific growth rate (SGR) = 100 × [(ln final body weight − ln initial body weight)/experimental days].

^5^Feed conversion ratio (FCR) = Dry feed fed/wet weight gain.

### 3.2. Early Intervention in Alevins Modulated Intermediary Metabolism in Adult Fish Fed a Linseed Oil‐Rich Challenge Diet

We examined the long‐term effects of LC‐PUFA injection on intermediary metabolism, including plasma metabolites and proximate composition of the liver, muscle, and whole body. Plasma triglyceride and total protein levels were significantly decreased in fish treated with linseed or fish oil injections compared with 0.85% NaCl (*p* < 0.05). Cholesterol tended to decrease in the linseed oil group (not significant) and was significantly decreased in the fish oil group (*p* < 0.05; Table 4). Plasma glucose, BUN, SGOT, and SGPT did not differ from control.

**Table 4:** Plasma metabolites of adult Nile tilapia (36 wpi) injected with saline (0.85% NaCl), linseed oil, or fish oil during the alevin stage and challenged with linseed oil‐rich diet during 37–40 wpi (mean ± SD, *n* = 6).

Plasma metabolites	0.85% NaCl history	Linseed oil history	Fish oil history	*p*‐Value
Glucose (mM)	4.73 ± 0.83	4.96 ± 0.68	4.00 ± 0.58	0.077
Triglyceride (mM)	3.29 ± 0.98^b^	1.85 ± 0.93^a^	1.46 ± 0.27^a^	**0.003**
Cholesterol (mM)	4.65 ± 0.78^b^	4.04 ± 0.43^a,b^	3.37 ± 0.67^a^	**0.013**
Total protein (mg/L)	35.06 ± 2.72^b^	30.36 ± 3.48^a^	30.48 ± 2.31^a^	**0.019**
BUN (mM)	0.96 ± 0.07	0.88 ± 0.12	0.93 ± 0.09	0.369
SGOT (U/L)	62.12 ± 5.95	57.16 ± 8.66	67.20 ± 5.90	0.073
SGPT (U/L)	40.50 ± 10.43	33.54 ± 7.42	31.73 ± 7.34	0.204

*Note:* Different superscript letters and bold values indicate significant differences in the mean values among experimental groups (*p* < 0.05).

Abbreviations: BUN, blood urea nitrogen; SGOT, serum glutamic oxaloacetic transaminase; SGPT, serum glutamate‐pyruvate transaminase; wpi, week postinjection.

Fish with linseed oil treatment exhibited higher hepatic fat content than that in the 0.85% NaCl treatment (*p* < 0.05; Table 5). Fish with fish oil treatment showed higher whole‐body protein than those in the 0.85% NaCl or linseed oil groups (*p* < 0.05). Notably, HSI, hepatic protein and ash content, muscle protein, fat, and ash content, as well as whole‐body fat and ash content, were similar among the experimental groups (Table 5).

**Table 5:** Proximate composition in the liver, muscles, and whole body of adult Nile tilapia (36 wpi) injected with saline (0.85% NaCl), linseed oil, or fish oil during the alevin stage and challenged with linseed oil‐rich diet at 37–40 wpi (mean ± SD, *n* = 6).

Proximate composition	0.85 % NaCl history	Linseed oil history	Fish oil history	*p*‐Value
Liver				
Protein (%)	15.60 ± 0.17	15.80 ± 0.24	15.87 ± 0.17	0.076
Fat (%)	4.48 ± 0.33^a^	5.27 ± 0.39^b^	4.87 ± 0.51^a,b^	**0.017**
Ash (%)	1.08 ± 0.05	1.07 ± 0.03	1.07 ±0.02	0.790
HSI (%)	3.21 ± 0.33	3.49 ± 0.17	3.51 ± 0.16	0.080
Muscle				
Protein (%)	21.08 ± 0.17	21.10 ± 0.18	21.00 ± 0.19	0.603
Fat (%)	1.99 ± 0.12	1.97 ± 0.11	1.99 ± 0.21	0.950
Ash (%)	1.13 ± 0.05	1.16 ± 0.03	1.13 ± 0.04	0.300
Whole body				
Protein (%)	16.41 ± 0.23^a^	16.35 ± 0.05^a^	16.68 ± 0.17^b^	**0.008**
Fat (%)	4.17 ± 0.35	4.79 ± 0.56	4.67 ± 0.31	0.056
Ash (%)	4.55 ± 0.13	4.66 ± 0.15	4.70 ± 0.22	0.283

*Note:* Different superscript letters and bold values indicate significant differences in the mean values among experimental groups (*p* < 0.05).

Abbreviations: HSI, hepatosomatic index; wpi, week postinjection.

### 3.3. Early Intervention in Alevins Modulated Fatty Acid Profiles in the Liver and Muscle of Adult Fish

PCA (Figure 2) provided a multivariate view of fatty acid profiles. In the liver, PC1 and PC2 explained 79.0% and 10.8% of variance, respectively (Figure 2), revealing three distinct clusters corresponding to 0.85% NaCl, linseed oil, and fish oil. Relative to 0.85% NaCl—and excluding C20:1*n*−9 (fish oil only) and C20:2 (linseed oil only)—both treatments significantly decreased several SFAs, MUFAs, *n*−3 LC‐PUFA, and *n*−6 LC‐PUFA, with more pronounced effects in the fish oil group (*p* < 0.05; Table 6). Notably, fish oil treatment significantly increased hepatic C20:3*n*−3 (*p* < 0.05).

Figure 2Principal component analysis (PCA) for fatty acid composition in the liver (89.8% of the total variance) (A) and muscle (81.8% of the total variance) (B) of adult Nile tilapia injected with saline (0.85% NaCl), linseed oil, or fish oil at the alevin stage and subsequently challenge with a linseed oil‐rich diet from 37 to 40 wpi. Different markers and colors represent stimulus history: 0.85% NaCl (green), linseed oil (orange), and fish oil (blue). *X*‐axis represents the variance explained by PC1 (liver; 79.0%, muscle; 51.8%), and *Y*‐axis represents the variance explained by PC2 (liver; 10.8%, muscle; 30.0%).

**Figure:**
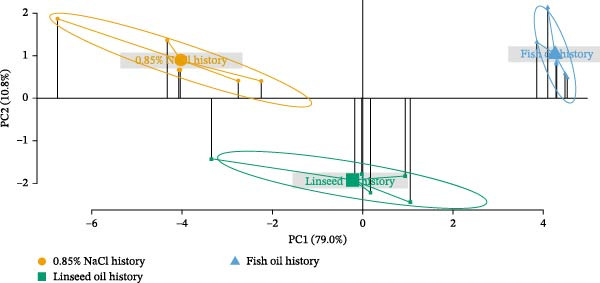
(A)

**Figure:**
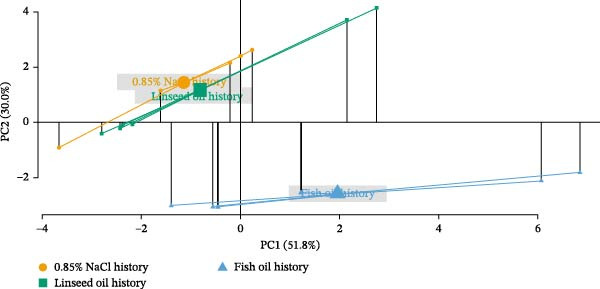
(B)

**Table 6:** Fatty acid composition in the liver of adult Nile tilapia injected with saline (0.85% NaCl), linseed oil, or fish oil during the alevin stage and challenged with linseed oil‐rich diet at 37–40 wpi (mean ± SD, *n* = 6).

Fatty acid (mg/100 g lipids)	0.85% NaCl history	Linseed oil history	Fish oil history	*p*‐Value
C8:0	0.28 ± 0.05^b^	0.17 ± 0.05^a^	0.20 ± 0.03^a^	**0.002**
C10:0	0.25 ± 0.06^b^	0.10 ± 0.03^a^	0.10 ± 0.02^a^	**<0.001**
C12:0	0.17 ± 0.09	0.18 ± 0.05	0.15 ± 0.02	0.817
C14:0	4.51 ± 0.63^c^	3.36 ± 0.71^b^	0.78 ± 0.10^a^	**<0.001**
C14:1	0.20 ± 0.03^c^	0.14 ± 0.03^b^	0.02 ± 0.00^a^	**<0.001**
C16:0	50.86 ± 6.82^c^	30.13 ± 6.71^b^	9.32 ± 1.00^a^	**<0.001**
C16:1	11.16 ± 1.56^c^	7.58 ± 1.64^b^	1.68 ± 0.21^a^	**<0.001**
C18:0	20.55 ± 2.73^c^	11.15 ± 2.59^b^	4.28 ± 0.46^a^	**<0.001**
C18:1*n*−9	63.95 ± 8.30^c^	36.10 ± 7.99^b^	13.56 ± 1.53^a^	**<0.001**
C18:2*n*−6	14.85 ± 1.59^c^	11.67 ± 2.41^b^	7.62 ± 1.11^a^	**<0.001**
C20:0	0.33 ± 0.04^c^	0.24 ± 0.06^b^	0.10 ± 0.01^a^	**<0.001**
C18:3*n*−6	0.92 ± 0.14^c^	0.51 ± 0.11^b^	0.10 ± 0.01^a^	**<0.001**
C20:1*n*−9	2.11 ± 0.22^a^	1.87 ± 0.33^a^	18.73 ± 3.17^b^	**<0.001**
C18:3*n*−3	3.14 ± 0.37^b^	1.63 ± 0.69^a^	1.39 ± 0.32^a^	**<0.001**
C18:4*n*−3	0.54 ± 0.10^c^	0.23 ± 0.05^b^	0.07 ± 0.01^a^	**<0.001**
C20:2	0.61 ± 0.09^b^	1.74 ± 0.26^c^	0.11 ± 0.01^a^	**<0.001**
C22:0	1.63 ± 0.23^c^	0.88 ± 0.19^b^	0.26 ± 0.04^a^	**<0.001**
C20:3*n*−6	1.73 ± 0.16^b^	1.47 ± 0.34^b^	0.11 ± 0.01^a^	**<0.001**
C22:1*n*−9	0.34 ± 0.05^c^	0.26 ± 0.06^b^	0.10 ± 0.01^a^	**<0.001**
C20:3*n*−3	0.39 ± 0.07^b^	0.27 ± 0.05^a^	0.74 ± 0.10^c^	**<0.001**
C20:4*n*−6	4.90 ± 0.81^c^	3.70 ± 0.95^b^	0.18 ± 0.06^a^	**<0.001**
C22:2	0.24 ± 0.04^c^	0.13 ± 0.05^b^	0.03 ± 0.00^a^	**<0.001**
C20:5*n*−3	0.27 ± 0.04^c^	0.20 ± 0.04^b^	0.05 ± 0.01^a^	**<0.001**
C24:0	0.41 ± 0.06^c^	0.27 ± 0.06^b^	0.05 ± 0.01^a^	**<0.001**
C24:1	1.10 ± 0.15^c^	0.70 ± 0.16^b^	0.22 ± 0.03^a^	**<0.001**
C22:6*n*−3	13.43 ± 1.88^c^	9.04 ± 1.84^b^	2.80 ± 0.36^a^	**<0.001**
SFA	79.23 ± 10.70^c^	46.67 ± 10.48^b^	15.30 ± 1.66^a^	**<0.001**
MUFA	79.51 ± 10.31^c^	47.01 ± 10.26^b^	34.41 ± 4.86^a^	**<0.001**
PUFA	41.24 ± 4.87^c^	30.78 ± 6.20^b^	14.27 ± 2.90^a^	**<0.001**
*n*−3 PUFA	17.77 ± 2.40^c^	11.37 ± 2.54^b^	6.04 ± 2.20^a^	**<0.001**
*n*−6 PUFA	22.62 ± 2.47^c^	17.54 ± 3.54^b^	8.09 ± 1.10^a^	**<0.001**
*n*−3/*n*−6 PUFA	0.78 ± 0.05	0.65 ± 0.07	0.74 ± 0.21	0.239
*n*−6/*n*−3 PUFA	1.28 ± 0.09^a^	1.56 ± 0.17^b^	1.48 ± 0.22^a,b^	**0.033**

*Note:* Different superscript letters and bold values indicate significant differences in the mean values among experimental groups (*p* < 0.05).

Abbreviations: MUFA, monounsaturated fatty acid; PUFA, polyunsaturated fatty acid; SFA, saturated fatty acid; wpi, week postinjection.

In the muscle, PC1 and PC2 explained 51.8% and 30.0% of variance, respectively (Figure 2B), again showing three clusters, with fish oil history most divergent. Both treatments significantly increased several fatty acids, including C8:0, C10:0, C12:0, *n*−3 PUFA, and the *n*−3/*n*−6 PUFA ratio (*p* < 0.05) compared to the 0.85% NaCl group. Each treatment induced specific changes (Table 7): linseed oil decreased C18:3*n*−6, C18:4*n*−3, and C22:6*n*−3, and increased C18:3*n*−3; fish oil increased C14:0 and C16:0 (raising total SFA), and—except for reduced C18:1*n*‐9 and C20:1*n*−9—increased C14:1, C16:1, C22:2, and C24:1. Although C18:4*n*−3 was decreased, *n*−3 LC‐PUFAs such as C20:3*n*−3 and C20:5*n*−3 were significantly elevated in the muscle of fish oil‐injected adults (*p* < 0.05). For *n*−6 LC‐PUFAs, fish oil history increased C20:4*n*−6 and decreased C18:2*n*−6, reducing total *n*−6 PUFA (*p* < 0.05).

**Table 7:** Fatty acid composition in the muscle of adult Nile tilapia injected with saline (0.85% NaCl), linseed oil, or fish oil during the alevin stage and challenged with linseed oil‐rich diet at 37–40 wpi (mean ± SD, *n* = 6).

Fatty acid (mg/100 g lipids)	0.85% NaCl history	Linseed oil history	Fish oil history	*p*‐Value
C8:0	0.11 ± 0.03^a^	0.71 ± 0.29^b^	0.59 ± 0.28^b^	**0.001**
C10:0	0.22 ± 0.07^a^	0.73 ± 0.29^b^	0.74 ± 0.37^b^	**0.006**
C12:0	0.19 ± 0.05^a^	0.56 ± 0.23^b^	0.52 ± 0.25^b^	**0.009**
C14:0	0.54 ± 0.12^a^	0.38 ± 0.15^a^	7.76 ± 2.69^b^	**<0.001**
C14:1	0.03 ± 0.01^a^	0.06 ± 0.02^a^	0.10 ± 0.05^b^	**0.005**
C16:0	13.92 ± 3.12^a^	11.85 ± 4.62^a^	23.12 ± 8.29^b^	**0.009**
C16:1	0.90 ± 0.20^a^	0.61 ± 0.24^a^	9.64 ± 3.25^b^	**<0.001**
C18:0	6.84 ± 1.50	6.33 ± 2.48	5.97 ± 2.20	0.774
C18:1*n*−9	26.25 ± 5.81^b^	25.37 ± 9.86^b^	25.78 ± 5.62^a^	**0.048**
C18:2*n*−6	22.07 ± 4.90^b^	21.72 ± 8.43^b^	3.86 ± 1.44^a^	**<0.001**
C20:0	0.39 ± 0.11	0.34 ± 0.13	0.50 ± 0.23	0.233
C18:3*n*−6	0.35 ± 0.15^b^	0.12 ± 0.04^a^	0.26 ± 0.09^b^	**0.006**
C20:1*n*−9	2.04 ± 0.60^b^	1.60 ± 0.75^a,b^	1.10 ± 0.35^a^	**0.044**
C18:3*n*−3	1.08 ± 0.19^a^	8.90 ± 3.19^b^	1.71 ± 0.58^a^	**<0.001**
C18:4*n*−3	0.71 ± 0.21^b^	0.39 ± 0.20^a^	0.25 ± 0.08^a^	**0.001**
C20:2	2.30 ± 0.51^a,b^	1.43 ± 0.59^a^	3.22 ± 1.14^b^	**0.005**
C22:0	0.33 ± 0.08^a,b^	0.21 ± 0.08^a^	0.44 ± 0.13^b^	**0.004**
C20:3*n*−6	0.22 ± 0.05	0.26 ± 0.10	0.21 ± 0.08	0.589
C22:1*n*−9	0.13 ± 0.03^a,b^	0.11 ± 0.05^a^	0.19 ± 0.07^b^	**0.049**
C20:3*n*−3	0.65 ± 0.14^a^	0.27 ± 0.12^a^	3.31 ± 1.12^b^	**<0.001**
C20:4*n*−6	1.25 ± 0.54^a^	1.32 ± 0.64^a^	4.05 ± 1.35^b^	**<0.001**
C22:2	0.03 ± 0.01^a^	0.03 ± 0.01^a^	0.05 ± 0.01^b^	**0.011**
C20:5*n*−3	0.73 ± 0.17^a^	0.48 ± 0.18^a^	1.90 ± 0.58^b^	**<0.001**
C24:0	0.17 ± 0.03	0.18 ± 0.07	0.17 ± 0.06	0.927
C24:1	0.14 ± 0.08^a^	0.10 ± 0.04^a^	0.69 ± 0.25^b^	**<0.001**
C22:6*n*−3	1.56 ± 0.36^b^	0.84 ± 0.35^a^	1.45 ± 0.45^b^	**0.013**
SFA	23.14 ± 5.18^a^	21.39 ± 8.34^a^	40.63 ± 14.73^b^	**0.009**
MUFA	29.59 ± 6.64	27.92 ± 10.99	28.40 ± 9.85	0.951
PUFA	31.14 ± 6.87^a,b^	35.91 ± 13.39^b^	20.56 ± 7.00^a^	**0.040**
*n*−3 PUFA	4.72 ± 0.93^a^	10.89 ± 3.90^b^	8.62 ± 2.81^b^	**0.006**
*n*−6 PUFA	24.10 ± 5.50^b^	23.57 ± 9.21^b^	8.67 ± 3.03^a^	**0.001**
*n*−3/*n*−6 PUFA	0.20 ± 0.01^a^	0.47 ± 0.10^b^	1.00 ± 0.03^c^	**<0.001**
*n*−6/*n*−3 PUFA	5.08 ± 0.25^c^	2.29 ± 0.50^b^	1.00 ± 0.03^a^	**<0.001**

*Note:* Different superscript letters and bold values indicate significant differences in the mean values among experimental groups (*p* < 0.05).

Abbreviations: MUFA, monounsaturated fatty acid; PUFA, polyunsaturated fatty acid; SFA, saturated fatty acid; wpi, week postinjection.

### 3.4. Early Intervention in Alevins Modulated Lipid Metabolic Pathways in the Liver and Muscle of Adult Fish

At the molecular level (Tables 8 and 9), linseed oil challenge diet downregulated hepatic *elovl7* and the fat‐transport gene *mttp* compared to the 0.85% NaCl group (*p* < 0.05). Fish oil treatment downregulated hepatic *pparα* (β‐oxidation), *fads2* (biosynthesis), and *mttp* (*p* < 0.05). There were no significant differences in *cpt1cb*, *acox1*, *elovl6*, *hmgcs1*, *mlxipl*, *acaca*, and *alox5*.

**Table 8:** mRNA expression levels of lipid metabolism‐related genes in the liver of adult Nile tilapia injected with saline (0.85% NaCl), linseed oil, or fish oil during the alevin stage and challenged with a linseed oil‐rich diet at 37–40 wpi (mean ± SD, *n* = 6).

	0.85% NaCl history	Linseed oil history	Fish oil history	*p*‐Value
Fatty acid β‐oxidation				
* cpt1cb*	1.13 ± 0.26	0.73 ± 0.19	1.15 ± 0.63	0.105
* acox1*	1.30 ± 0.39	0.84 ± 0.13	1.07 ± 0.58	0.181
* pparα*	1.25 ± 0.40^a^	1.00 ± 0.36^a,b^	0.71 ± 0.17^b^	**0.036**
Fatty acid biosynthesis				
* fads2*	1.46 ± 0.58^a^	1.39 ± 0.26^a^	0.53 ± 0.23^b^	**0.003**
* elovl6*	1.12 ± 0.65	0.84 ± 0.54	1.10 ± 1.06	0.793
* elovl7*	3.67 ± 1.09^a^	2.35 ± 0.51^b^	3.34 ± 0.73^a,b^	**0.033**
Fat transportation				
* mttp*	1.30 ± 0.37^a^	0.90 ± 0.17^b^	0.86 ± 0.31^b^	**0.041**
Cholesterol metabolism				
* hmgcs1*	1.22 ± 0.40	0.81 ± 0.14	1.02 ± 0.42	0.183
Interaction between carbohydrate and lipid metabolism				
* mlxipl*	1.33 ± 0.26	0.87 ± 0.14	1.11 ± 0.40	0.085
* acaca*	1.28 ± 0.43	0.72 ± 0.13	0.98 ± 0.58	0.160
Eicosanoid synthesis				
* alox5*	5.59 ± 2.76	2.78 ± 1.06	4.53 ± 3.35	0.196

*Note:* Different superscript letters and bold values indicate significant differences in the mean values among experimental groups (*p* < 0.05).

Abbreviation: wpi, week postinjection.

**Table 9:** mRNA expression levels of lipid metabolism‐related genes in the muscle of adult Nile tilapia injected with saline (0.85% NaCl), linseed oil, or fish oil during the alevin stage and challenged with a linseed oil‐rich diet at wpi 37–40 (mean ± SD, *n* = 6).

	0.85% NaCl history	Linseed oil history	Fish oil history	*p*‐Value
Fatty acid β‐oxidation				
* cpt1cb*	0.84 ± 0.20^b^	1.04 ± 0.18^a,b^	1.31 ± 0.29^a^	**0.010**
* acox1*	0.81 ± 0.23	0.79 ± 0.39	1.21 ± 0.52	0.156
* pparα*	0.68 ± 0.13^b^	0.78 ± 0.29^a,b^	1.09 ± 0.25^a^	**0.028**
Fatty acid biosynthesis				
* Fads2*	0.89 ± 0.26	0.65 ± 0.17	0.81 ± 0.14	0.135
* elovl6*	0.50 ± 0.17^c^	0.77 ± 0.12^b^	1.34 ± 0.27^a^	**<0.001**
* elovl7*	0.59 ± 0.23^b^	0.73 ± 0.19^b^	1.20 ± 0.42^a^	**0.008**
Fat transportation				
* mttp*	0.56 ± 0.18^b^	0.73 ± 0.15^b^	1.33 ± 0.30^a^	**<0.001**
Cholesterol metabolism				
* hmgcs1*	0.74 ± 0.25	0.82 ± 0.45	1.32 ± 0.57	0.078
Interaction between carbohydrate and lipid metabolism				
* mlxipl*	0.78 ± 0.11^b^	1.65 ± 0.38^a^	1.73 ± 0.24^a^	**<0.001**
* acaca*	0.86 ± 0.14^b^	0.91 ± 0.26^b^	1.31 ± 0.33^a^	**0.015**
Eicosanoid synthesis				
* alox5*	0.76 ± 0.23	0.98 ± 0.40	1.42 ± 0.89	0.173

*Note:* Different superscript letters and bold values indicate significant differences in the mean values among experimental groups (*p* < 0.05).

Abbreviation: wpi, week postinjection.

In the muscle, linseed oil treatment upregulated *elovl6* and *mlxipl* (*p* < 0.05), while fish oil treatment upregulated *cpt1cb*, *pparα*, *elovl6*, *elovl7*, *mttp*, *mlxipl*, and *acaca* (*p* < 0.05). However, *acox1*, *fads2*, *hmgcs1*, and *alox5* were unchanged.

### 3.5. Early Intervention in Alevins Reversed the Altered Expression of Enzymes Related to DNA Methylation and Histone Modification in Adult Fish Fed a Linseed Oil‐Rich Diet

Table 10 shows hepatic gene expression related to DNA methylation and histone modifications. Linseed oil treatment significantly induced *dnmt3aa* (*p* < 0.05) compared to 0.85% NaCl. Both linseed and fish oil treatments upregulated *tet1*, *tet2*, and *tet3* (*p* < 0.05). For histone‐modification‐related genes, hepatic expression of H3K4me3 writers (*setd1a* and *setd1ba*), H3K4me3 erasers (*kdm5bb*), H3K9ac writers (*kat2b* and *gtf3c4*), and H3K9ac erasers (*sirt2* and *sirt5*) was upregulated by both treatments (*p* < 0.05). Further, linseed oil treatment upregulated *kmt2ba*, *kdm5c*, *riox1*, and *kat6a* (*p* < 0.05).

**Table 10:** mRNA expression levels of genes involved in the hepatic epigenetic landscape of adult Nile tilapia injected with saline (0.85% NaCl), linseed oil, or fish oil during the alevin stage and challenged with a linseed oil‐rich diet at 37–40 wpi (mean ± SD, *n* = 6).

	0.85% NaCl history	Linseed oil history	Fish oil history	*p*‐Value
DNA methylation writer				
* dnmt3aa*	0.48 ± 0.15^b^	0.87 ± 0.20^a^	0.65 ± 0.17^a,b^	**0.011**
* dnmt3bb*	0.71 ± 0.29	1.72 ± 1.52	1.13 ± 0.57	0.216
DNA methylation eraser				
* tet1*	0.42 ± 0.13^b^	1.02 ± 0.23^a^	0.76 ± 0.19^a^	**<0.001**
* tet2*	0.49 ± 0.20^b^	1.12 ± 0.25^a^	0.85 ± 0.20^a^	**0.001**
* tet3*	0.53 ± 0.24^b^	1.19 ± 0.20^a^	0.91 ± 0.19^a^	**<0.001**
H3K4me3 writer				
* kmt2ba*	0.38 ± 0.18^b^	1.18 ± 0.55^a^	0.84 ± 0.21^a,b^	**0.008**
* kmt2bb*	0.43 ± 0.14	1.40 ± 0.94	1.20 ± 0.89	0.090
* setd1a*	0.39 ± 0.15^b^	1.07 ± 0.30^a^	0.81 ± 0.28^a^	**0.001**
* setd1ba*	0.40 ± 0.17^b^	1.11 ± 0.29^a^	0.79 ± 0.24^a^	**<0.001**
H3K4me3 eraser				
* kdm5ba*	0.36 ± 0.15	0.66 ± 0.27	0.50 ± 0.18	0.067
* kdm5bb*	0.43 ± 0.09^b^	0.92 ± 0.22^a^	0.74 ± 0.10^a^	**<0.001**
* kdm5c*	0.51 ± 0.21^b^	1.08 ± 0.27^a^	0.85 ± 0.35^a,b^	**0.012**
* riox1*	0.48 ± 0.24^b^	1.20 ± 0.29^a^	0.77 ± 0.28^b^	**0.001**
H3K9ac writer				
* kat2b*	0.45 ± 0.18^c^	1.08 ± 0.21^a^	0.76 ± 0.20^b^	**<0.001**
* kat6a*	0.50 ± 0.19^b^	1.79 ± 0.79^a^	0.91 ± 0.24^b^	**0.002**
* gtf3c4*	0.72 ± 0.28^c^	2.08 ± 0.56^a^	1.28 ± 0.24^b^	**0.001**
H3K9ac eraser				
* sirt2*	0.39 ± 0.14^b^	0.98 ± 0.30^a^	0.73 ± 0.24^a^	**0.002**
* sirt5*	0.53 ± 0.22^c^	1.19 ± 0.24^a^	0.84 ± 0.21^b^	**0.001**

*Note:* Different superscript letters and bold values indicate significant differences in the mean values among experimental groups (*p* < 0.05).

Abbreviation: wpi, week postinjection.

Expression of hepatic *riox1*, *kat2b*, *gtf3c4*, *kat6a*, and *sirt5* was significantly higher in the linseed oil group than that in the fish oil group (*p* < 0.05; Table 10). No significant differences were observed for *dnmt3bb*, *kmt2bb*, and *kdm5ba*. In the muscle, no significant differences were detected for genes related to DNA methylation or histone modifications among groups (Table 11).

**Table 11:** mRNA expression levels of genes involved in the muscular epigenetic landscape of adult Nile tilapia injected with saline (0.85% NaCl), linseed oil, or fish oil during the alevin stage and challenged with a linseed oil‐rich diet at 37–40 wpi (mean ± SD, *n* = 6).

	0.85% NaCl history	Linseed oil history	Fish oil history	*p*‐Value
DNA methylation writer				
* dnmt3aa*	1.04 ± 0.31	1.06 ± 0.16	1.05 ± 0.18	0.979
* dnmt3bb*	1.09 ± 0.30	0.93 ± 0.18	1.07 ± 0.16	0.424
DNA methylation eraser				
* tet1*	1.29 ± 0.41	1.37 ± 0.26	1.38 ± 0.20	0.874
* tet2*	1.42 ± 0.43	1.60 ± 0.29	1.73 ± 0.20	0.285
* tet3*	1.67 ± 0.49	1.92 ± 0.41	2.14 ± 0.41	0.207
H3K4me3 writer				
* kmt2ba*	0.44 ± 0.16	0.49 ± 0.10	0.47 ± 0.18	0.844
* kmt2bb*	1.22 ± 0.41	1.49 ± 0.22	1.33 ± 0.29	0.364
* setd1a*	1.43 ± 0.56	1.62 ± 0.35	1.54 ± 0.29	0.741
* setd1ba*	1.07 ± 0.29	1.26 ± 0.31	1.22 ± 0.25	0.491
H3K4me3 eraser				
* kdm5bb*	1.81 ± 0.57	1.69 ± 0.26	1.56 ± 0.23	0.562
* kdm5c*	1.61 ± 0.41	1.66 ± 0.28	1.46 ± 0.24	0.553
* riox1*	1.32 ± 0.38	1.27 ± 0.16	1.15 ± 0.18	0.521
H3K9ac writer				
* kat2b*	2.18 ± 0.45	2.62 ± 0.32	2.27 ± 0.17	0.106
* kat6a*	1.87 ± 0.63	1.88 ± 0.36	1.83 ± 0.11	0.838
* gtf3c4*	1.35 ± 0.51	1.58 ± 0.54	1.54 ± 0.34	0.672
H3K9ac eraser				
* sirt2*	1.37 ± 0.35	1.33 ± 0.18	1.21 ± 0.10	0.501
*sirt5*	1.22 ± 0.20	1.36 ± 0.29	1.20 ± 0.12	0.400

Abbreviation: wpi, week postinjection.

## 4. Discussion

Nutritional stimulation by specific fatty acids during early life has been shown to exert long‐term effects on lipid metabolism, as well as on fatty acid utilization and deposition in marine fish such as gilthead seabream and seabass. These effects enhance the efficient use of diets with low LC‐PUFA content and modulate tissue fatty acid accumulation [12, 36]. In this study, we observed that linseed and fish oil injections into the yolk reserves—sources of *n*−3 PUFA and *n*−3 LC‐PUFA, respectively—led to increased levels of SFA, MUFA, *n*−3 PUFA, *n*−6 PUFA, and the *n*−3/*n*−6 PUFA ratio in fry at 1 wpi. Among the elevated fatty acids, C18:3*n*−3 levels were significantly increased in linseed oil‐injected fry, whereas C20:5*n*−3 and C22:6*n*−3 levels were elevated in fish oil‐injected fry, demonstrating the effectiveness of early‐life *n*−3 PUFA and *n*−3 LC‐PUFA stimulation in modulating the fatty acid profiles of Nile tilapia. PCA revealed three distinct clusters of hepatic and muscular fatty acid compositions in adults, corresponding to the 0.85% NaCl‐, linseed oil‐, and fish oil‐injected groups, suggesting that NP influenced lipid metabolism and fatty acid composition in the long term. NP of carbohydrate metabolism was previously achieved in Nile tilapia by glucose injection into the yolk reserve at the alevin stage, which induced long‐term modulatory effects on carbohydrate metabolism in adult fish [23, 24]. Similarly, NP via linseed oil injection into the yolk reserve of Nile tilapia alevins modulated several lipid metabolic responses, resulting in long‐term effects on fatty acid composition, fatty acid β‐oxidation, and LC‐PUFA biosynthesis, but not on growth during the juvenile stage [25]. Here, we demonstrated that the NP effects of linseed oil (*n*−3 PUFA) enrichment at the alevin stage persisted into adulthood, modulating both consistent and distinct lipid metabolic pathways when challenged with a linseed oil‐rich diet. We further examined the comparative NP effects of early fish oil (*n*−3 LC‐PUFA) injection on lipid metabolism in adult fish. Moreover, our findings suggest that these long‐term NP effects may be associated with epigenetic regulation.

### 4.1. Linseed Oil Injection at the Alevin Stage Improved Growth in Adult Fish Fed a Linseed Oil‐Rich Diet

Previous studies in mammals have shown that maternal rats fed a linseed oil‐rich diet during early pregnancy (0–12 days) produce male offspring with significantly higher body weights at 12 months of age compared to offspring of dams fed a fish oil‐rich diet [37]. Thus, maternal dietary lipid composition can influence fat accumulation in the adipose tissue of adult male offspring, contributing to long‐term increases in body weight. In our study, although linseed oil injection had no effect on growth during feeding with a commercial diet (up to 36 wpi), significant increases in body weight, weight gain, and ADG were observed in adult Nile tilapia challenged with a linseed oil‐rich diet (weeks 37–40). Notably, this weight gain during the challenge period did not significantly improve SGR or relative growth rate. Hence, NP via early linseed oil exposure may enhance the utilization efficiency of a linseed oil‐rich diet in adult Nile tilapia, thereby promoting absolute weight gain, thus demonstrating the potential benefits of NP with *n*−3 PUFA for improving the utilization of dietary *n*−3 PUFA.

However, a previous study showed that linseed oil injection into the yolk reserves of Nile tilapia larvae did not improve growth on a linseed oil‐rich diet during the juvenile stage [25], suggesting that the beneficial effects of NP may depend on the developmental phase. This could reflect stage‐specific differences in dietary *n*−3 PUFA utilization efficiency across the fish life cycle. For instance, juvenile yellowfin tuna primarily rely on protein as an energy source, whereas adults preferentially utilize lipids [38]. In summary, the long‐term effects of early‐life fatty acid programming on growth responses in fish may depend on developmental stage, possibly related to the capacity for lipid utilization.

### 4.2. Linseed and Fish Oil Injection at the Alevin Stage Exerted Long‐Term Effects on Intermediary Metabolism in Adult Fish

In mammals, early‐life exposure to diets rich in *n*−3 LC‐PUFA during prenatal and postnatal periods can exert long‐term effects on intermediary metabolism extending into adulthood [37, 39]. In rats, maternal intake of a linseed oil‐rich diet significantly increases lumbar adipose tissue weight in adult male offspring compared with offspring of dams fed a fish oil‐rich diet [37]. In the present study, under dietary challenge with a linseed oil‐rich diet, both linseed and fish oil injection treatments resulted in decreased plasma triglyceride levels and, in the case of fish oil only, reduced cholesterol levels. Additionally, linseed oil injection led to increased hepatic fat content, although not in muscle, accompanied by a reduction in plasma triglycerides. These findings suggest that early‐life exposure to *n*−3 PUFA and *n*−3 LC‐PUFA has lasting effects into adulthood, potentially reducing plasma lipemia while promoting hepatic fat accumulation by decreasing fat transport from the liver to the plasma. Indeed, downregulation of *mttp* expression was observed in hepatic tissue. Conversely, the opposite trend in *mttp* expression occurred in the muscle, without any corresponding change in muscle fat content. In mice, hepatic *mttp* inactivation reduces plasma triglyceride and cholesterol concentrations [40], and an inverse correlation exists between hepatic *mttp* mRNA expression and hepatic steatosis severity [41]. These results differ from those in juvenile fish, where linseed oil injection decreased hepatic fat and upregulated *mttp* expression [25]. In our study, plasma protein levels were reduced in both linseed oil‐ and fish oil‐injected groups, while an increase in whole‐body protein content was observed only in the fish oil group, suggesting that early fish oil exposure may promote tissue protein deposition in adulthood. These findings are in contrast with those in juveniles [25]. In gilthead sea bream, a carnivorous species, feeding broodstock an *n*−3 PUFA‐rich diet for 3 months did not affect hepatic or muscular nutrient composition in 16‐month‐old offspring [12]. In summary, the NP effects of early exposure to *n*−3 PUFA and *n*−3 LC‐PUFA on intermediary metabolism may vary depending on developmental stage, fish species, and trophic habit.

### 4.3. Linseed and Fish Oil Injection at the Alevin Stage Modulated Lipid Metabolism and Associated Pathways in Adult Fish

Fatty acid interventions during critical developmental stages influence lipid metabolism and fatty acid composition in fish over the long term, though outcomes vary among species. For example, in gilthead sea bream, dietary LC‐PUFA stimulation in broodstock affects liver fatty acid composition in juvenile offspring challenged with reminder diets [10, 42]. In Nile tilapia, linseed oil overload during the alevin stage increases hepatic and muscular levels of several SFAs, *n*−6 PUFA, and *n*−3 PUFA during the juvenile stage under a linseed oil‐rich dietary challenge [25]. However, in the present study, adult fish challenged with the same diet showed reduced hepatic levels of several SFAs, *n*−6 PUFA, and *n*−3 PUFA following early linseed oil injection. Thus, the long‐term NP effects of early *n*−3 PUFA overload on hepatic fatty acid deposition—where the liver serves as the primary site for *n*−3 PUFA utilization and storage—differ between juvenile and adult stages. In contrast, similar trends in muscle fatty acid responses to early *n*−3 PUFA interventions were observed in both stages [25]. Early linseed oil injection (rich in C18:3*n*−3) increased muscular C18:3*n*−3 levels, while fish oil injection (rich in C20:5*n*−3 and C22:6*n*−3) increased C20:3*n*−3, C20:4*n*−6, and C20:5*n*−3. Both interventions elevated total *n*−3 PUFA and the *n*−3/*n*−6 PUFA ratio in muscle. Therefore, early‐life overload of *n*−3 PUFA and *n*−3 LC‐PUFA can promote their deposition in the muscle at the adult stage. NP with *n*−3 PUFA and *n*−3 LC‐PUFA may, thus, serve as an effective strategy to enhance muscle fatty acid profiles and meat quality in aquaculture.

The effects of early NP with dietary lipids on tissue‐specific fatty acid β‐oxidation depend on the lipid source and developmental stage. Previous studies in juvenile Nile tilapia showed that linseed oil injection upregulated hepatic *cpt1cb*, *acox1*, and *pparα*, indicating enhanced β‐oxidation [25]. In contrast, in adults, linseed oil injection suppressed hepatic *pparα*, suggesting inhibition of peroxisomal β‐oxidation. This difference highlights distinct NP effects of early linseed versus fish oil exposure. Similarly, in gilthead seabream, fish oil‐fed broodstock produced offspring with higher hepatic *cpt1* expression than linseed oil‐fed brood stock [12]. In European sea bass, larvae fed low‐HUFA diets exhibited higher *pparα* expression than those fed a high‐HUFA diet, with sustained induction later in juveniles [36]. The role of β‐oxidation also differs between the liver and the muscle: the liver generates ketone bodies for other tissues, while muscle oxidizes fatty acids for its own energy utilization. Consistent with this, fish oil‐injected adults showed increased muscular β‐oxidation (*cpt1cb* and *pparα* upregulation), whereas linseed oil‐injected fish did not.

In juvenile Nile tilapia, early linseed oil injection upregulated hepatic and muscular fatty acid desaturation (*fads2*) and elongation (*elovl6*) genes while downregulating muscular *elovl7* [25]. This modulation likely contributed to elevated hepatic *n*−3 LC‐PUFA (EPA and DHA). In contrast, in adults, linseed oil injection downregulated hepatic *elovl7*, potentially reducing *n*−3 LC‐PUFA levels, while upregulated muscular *elovl6* may explain the increase in *n*−3 PUFA. Thus, NP effects vary by tissue and developmental stage.

Dietary fish oil suppresses hepatic desaturase and elongase activity in several species [36, 43–45]. Consistently, early fish oil injection reduced hepatic *fads2* expression and decreased *n*−3 and *n*−6 LC‐PUFA levels in adulthood. However, *elovl6* and *elovl7* were upregulated in the muscle, corresponding to increased muscular LC‐PUFA deposition. Both early linseed and fish oil injections increased total *n*−3 PUFA and the *n*−3/*n*−6 PUFA ratio in the muscle, supporting NP’s application for enhancing fish meat quality.

Our study further revealed that early linseed oil injection induced *mlxipl* expression in the muscle, but not in the liver, while fish oil injection upregulated both *mlxipl* and *acaca*, in contrast to a previous study [25]. In mammals, inhibition of *mlxipl* promotes fatty acid β‐oxidation and reduces lipid content [46]. Thus, early *n*−3 PUFA and *n*−3 LC‐PUFA exposure may influence carbohydrate metabolism in adult Nile tilapia via modulation of *mlxipl* and *acaca*, potentially suppressing muscular gluconeogenesis.

The long‐term NP effects of early linseed oil injection on several lipid‐related pathways observed in juvenile fish [25] were not maintained in adults. For example, altered *mttp* and *alox5* levels in juveniles were not evident in adults, indicating that NP‐induced stimulation of lipid transport and eicosanoid synthesis may diminish with age. Notably, fish oil injection did not affect *alox5* expression at either stage.

### 4.4. Linseed and Fish Oil Injection at the Alevin Stage Affected DNA Methylation and Histone Modifications in Adult Fish

Epigenetic regulation is proposed as a mechanism underlying NP‐mediated long‐term metabolic modulation. In mammals, fatty acid–based nutritional interventions during parental or early developmental stages can induce lasting epigenetic changes through DNA methylation and histone modifications [47, 48]. DNA methylation dynamics involve “writers” (methyltransferases) and “erasers” (demethylases), that play key roles in nutri‐epigenetics [49]. For example, feeding female rats or lactating mice a high‐SFA diet downregulates *dnmt1* and upregulates *tet1* expression in the adipose tissue of male offspring, with effects persisting into adulthood [50, 51]. Similarly, low maternal *n*−3 PUFA intake induces hypermethylation of the brain‐derived neurotrophic factor gene, impairing brain development in adult offspring [52]. In this study, early linseed oil and fish oil injections modulated epigenetic markers in the liver—but not in the muscle—with stronger effects observed with linseed oil. Specifically, linseed oil injection upregulated *dnmt3aa* (a DNA methylation writer) and both interventions upregulated *tet1*, *tet2*, and *tet3* (demethylation‐related genes). These findings indicate that the liver is the primary site of epigenetic programming and that early *n*−3 PUFA and *n*−3 LC‐PUFA exposure influences DNA (de)methylation through both writers and erasers. Dietary lipids alter DNA methylation in aquatic species; for instance, LC‐PUFA‐enriched diets slightly increase global DNA methylation in *Octopus vulgaris* [53], and long‐term *n*−3 LC‐PUFA intake induces methylated CpG sites [54]. DHA and EPA promote promoter demethylation of *cox2* and *ppar*γ in colorectal cancer cells [55]. Thus, *n*−3 PUFAs and *n*−3 LC‐PUFAs may modulate DNA (de)methylation to induce epigenetic programming that persists into adulthood and modulates lipid metabolism, particularly under *n*−3 PUFA‐rich diets.

Histone modification is another key epigenetic mechanism influencing chromatin accessibility and transcription. Histone lysine methyltransferases (e.g., *KMT2*) and demethylases (*KDM* family) regulate histone methylation, while acetyltransfereases (e.g., *KAT2B* and *Gtf3c4*) and histone deacetylases (e.g., *SIRT2* and *SIRT5*) control histone acetylation [56–62]. NP effects of dietary fatty acids can alter these regulators: in mammals, SFA‐ and *n*−3 PUFA‐rich maternal diets reduce hepatic SIRT1, HDAC1, and HAT activities in offspring [48, 63]. In this study, early linseed and fish oil injections modulated histone‐modification enzymes in the liver, with stronger effects observed with linseed oil. In the linseed oil‐injected group, upregulated genes included H3K4me3 writers (*kmt2ba*, *setd1a*, and *setd1ba*), erasers (*kdm5bb*, *kdm5c*, and *riox1*), H3K9ac writers (*kat2b*, *kat6a*, and *gtf3c4*), and erasers (*sirt2* and *sirt5*). In the fish oil‐injected group, H3K4me3 writers (*setd1a* and *setd1ba*), erasers (*kdm5bb*), H3K9ac writers (*kat2b* and *gtf3c4*), and erasers (*sirt2* and *sirt5*) were upregulated. These results suggest that histone modifications in the liver form a dynamic regulatory system influenced by early‐life exposure to *n*−3 PUFA and *n*−3 LC‐PUFA. Dietary combinations such as fish oil and pectin have been shown to induce global histone state changes, upregulating genes involved in lipid catabolism and β‐oxidation [64]. Taken together, early *n*−3 PUFA or *n*−3 LC‐PUFA stimulation may drive epigenetic programming that persists into adulthood and modulates lipid metabolism through coordinated DNA (de)methylation and histone modification in the liver.

## 5. Conclusion

The long‐term NP effects of *n*−3 PUFA and *n*−3 LC‐PUFA administered via linseed oil and fish oil injections, respectively, during the alevin stage persisted into adulthood in Nile tilapia, influencing lipid metabolism and related pathways. Following dietary challenge with a linseed oil‐rich diet, fish with a history of linseed oil injection exhibited improved efficiency in utilizing *n*−3 PUFA for weight gain. Early exposure to fish oil led to tissue‐specific modulation of fatty acid β‐oxidation, characterized by decreased hepatic and increased muscular β‐oxidation. With more pronounced effects with a history of *n*−3 LC‐PUFA injection, overload of *n*−3 PUFA and *n*−3 LC‐PUFA during early development reduced plasma lipemia while promoting hepatic fat accumulation, decreased hepatic but increased muscular LC‐PUFA deposition and influenced lipid‐related metabolic processes. Furthermore, epigenetic regulation particularly involving DNA methylation and histone modifications appeared to contribute to these long‐term NP effects.

**Nomenclature**
BUN: Blood urea nitrogen
CF: Crude fat
CHO: Carbohydrate
CP: Crude protein
DHA: Docosahexaenoic acid
DO: Dissolved oxygen
EFAs: Essential fatty acids
EPA: Eicosapentaenoic acid
FAME: Fatty acid methyl ester
HSI: Hepatosomatic index
LC‐PUFA: Long‐chain polyunsaturated fatty acid
MUFA: Monounsaturated fatty acid
NP: Nutritional programming
PCA: Principal component analysis
qRT‐PCR: Real‐time reverse transcription PCR
SFA: Saturated fatty acid
SGOT: Serum aspartate aminotransferase
SGPT: Serum alanine aminotransferase
wpi: Week postinjection.

## Author Contributions

**Linli Luo**: first draft preparation, data curation. **Sirijanya Thongchaitriwat**: methodology, data curation. **Suksan Kumkhong**: fish culture. **Janethida Kiatmontri**: data analysis. **Shenglin Yang**: conceptualization. **Stephane Panserat and Surintorn Boonanuntanasarn**: conceptualization, writing – reviewing and editing.

## Funding

This project was supported by the National Research Council of Thailand (NRCT) (Grant N42A650324) and Suranaree University of Technology (SUT).

## Disclosure

All authors have read and approved the final manuscript.

## Ethics Statement

All experimental protocols were approved by the Ethics Committee of Suranaree university of Technology, Animal care and Use Committee (Approval Number A‐18/2562).

## Conflicts of Interest

The authors declare no conflicts of interest.

## Supporting Information

Additional supporting information can be found online in the Supporting Information section.

## Supporting information


**Supporting Information 1** Survival rate of all experimental fry through 5 wpi after sex reversal.

**Supporting Information 2** List of qRT‐PCR primers for lipid metabolism‐related genes in Nile tilapia.

**Supporting Information 3** List of qRT‐PCR primers of genes related to epigenetics in Nile tilapia.

## Data Availability

The data that support the findings of this study are available from the corresponding author upon reasonable request.
