# Supplementary material for: The Injection of Lipids Into Yolk Sac has Been Shown to Alter the Lipid Metabolism of Adult Nile Tilapia (Oreochromis niloticus)
Source: Aquac Nutr. 2026 Feb 25;2026:8360989. doi: 10.1155/anu/8360989 (PMC12933634; doi:10.1155/anu/8360989)
Supplement: Supplementary file 1 — Supporting Information 1 Survival rate of all experimental fry through 5 wpi after sex reversal. [file ANU-2026-8360989-s002.pdf]

Aquaculture Nutrition

Supplementary information

Supporting Information 1: Figure S1

The injection of lipids into yolk sac has been shown to alter the lipid metabolism of adult Nile tilapia (*Oreochromis niloticus*)

Linli Luo[a], Sirijanya Thongchaitriwat[a], Suksan Kumkhong[a], Janethida Kiatmontri[a], Shenglin Yang[b], Stephane Panserat[c], Surintorn Boonanuntanasarn[a],*

[a] *School of Animal Technology and Innovation, Institute of Agricultural Technology, Suranaree University of Technology, Nakhon Ratchasima, Thailand 30000*

[b] *Key Laboratory of Animal Genetics, Breeding and Reproduction in the Plateau Mountainous Region, Ministry of Education, Guizhou University, Guiyang, 550025, China*

[c] *National Research Institute for Agriculture Food and Environment, Université de Pau & Pays de L'Adour, NuMeA, Aquapôle, 64310 Saint-Pée-Sur-Nivelle, France.*

* Corresponding author: surinton@sut.ac.th (Surintorn Boonanuntanasarn)

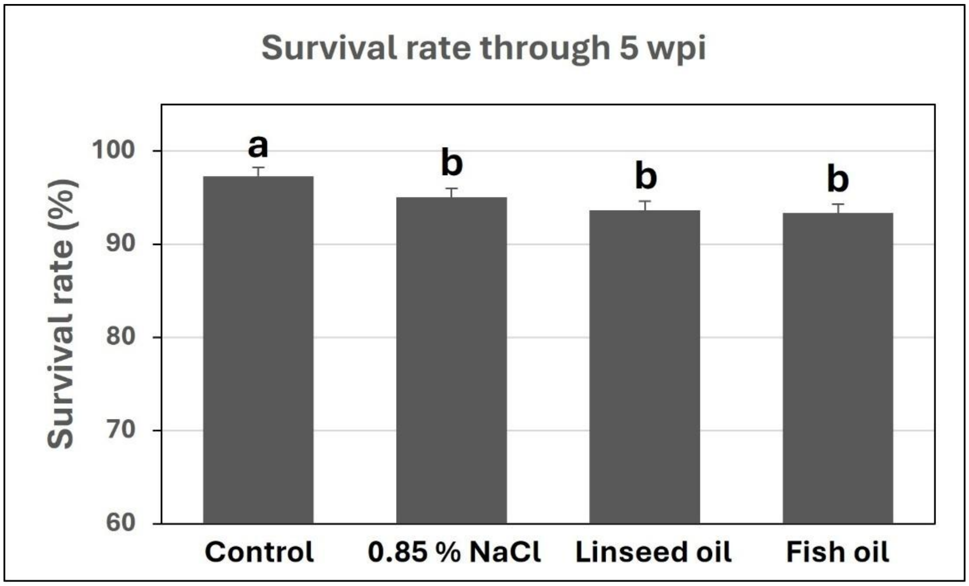

Figure S1. Survival rate of all experimental fry through 5 wpi after sex reversal.
