# Supplementary material for: The Injection of Lipids Into Yolk Sac has Been Shown to Alter the Lipid Metabolism of Adult Nile Tilapia (Oreochromis niloticus)
Source: Aquac Nutr. 2026 Feb 25;2026:8360989. doi: 10.1155/anu/8360989 (PMC12933634; doi:10.1155/anu/8360989)
Supplement: Supplementary file 2 — Supporting Information 2 List of qRT‐PCR primers for lipid metabolism‐related genes in Nile tilapia. [file ANU-2026-8360989-s003.pdf]

Aquaculture Nutrition

Supplementary information

Supporting Information 2: Table S1

The injection of lipids into yolk sac has been shown to alter the lipid metabolism of adult Nile tilapia (*Oreochromis niloticus*)

Linli Luo[a], Sirijanya Thongchaitriwat[a], Suksan Kumkhong[a], Janethida Kiatmontri[a], Shenglin Yang[b], Stephane Panserat[c], Surintorn Boonanuntanasarn[a],*

[a] *School of Animal Technology and Innovation, Institute of Agricultural Technology, Suranaree University of Technology, Nakhon Ratchasima, Thailand 30000*
[b] *Key Laboratory of Animal Genetics, Breeding and Reproduction in the Plateau Mountainous Region, Ministry of Education, Guizhou University, Guiyang, 550025, China*
[c] *National Research Institute for Agriculture Food and Environment, Université de Pau & Pays de L'Adour, NuMeA, Aquapôle, 64310 Saint-Pée-Sur-Nivelle, France.*

* Corresponding author: surinton@sut.ac.th (Surintorn Boonanuntanasarn)

SUPPLEMENTARY TABLE S1: List of qRT-PCR primers for lipid metabolism-related genes in Nile tilapia

Genes	5'/3' Forward primer	5'/3' Reverse primer	Accession numbers
Reference gene			
*ef1**	GCACGCTCTGCTGGCCTTT	GCGCTCAATCTTCCATCCC	AB075952.1
Fatty acid β-oxidation			
cpt1cb	GGATGACGAGGAGCAAGGCA	GGTGCATCAGCCCAGGAGTG	XM_003446465.5
acox1	GGTGGCCACGGTTACTCTCG	ACGACACAATGCCACTCAGC	NM_001290199.1
pparα	GCCCTCTTCACCCTCTTGGC	AGCTAGGTCGCTGTCGTCCA	XM_025899322.1
Fatty acid biosynthesis			
fads2	ATCTGGCGTGGTCCTTGTCG	ACTGCATGGTCAGCCAGTCC	XM_025908789.1
elovl6	CTGGCGGTGGGTGGTTCAT	CCCTGCTGCATCCACGAGTA	XM_003443399.5
elovl7	TGACCAGCCACATCTCCCAGT	TGGTGCGACCATGTCTGAGC	XM_003446137.5
Fat transportation			
mttp	TCCTGCCTGAGGCCATTCCT	GCAGCGGCAGCTCTCACATT	XM_005458713.4
Interaction between carbohydrate and lipid metabolism			
mlxipl	TCTGCAAAGCCAGAGTGCCA	GCTGCTCCTGTCAGTGCTGT	XM_019354389.2
acaca	TTGAGGATGGCGGGCATGTG	CTGCTGCACTCTGCTTGGGT	XM_031730686.2
Eicosanoid synthesis			
alox5	CTGATGCTGTGGGAGGCCAT	ACTGCCAGCAGTGTGCAGAG	XM_003458399.5
