# Supplementary material for: The Injection of Lipids Into Yolk Sac has Been Shown to Alter the Lipid Metabolism of Adult Nile Tilapia (Oreochromis niloticus)
Source: Aquac Nutr. 2026 Feb 25;2026:8360989. doi: 10.1155/anu/8360989 (PMC12933634; doi:10.1155/anu/8360989)
Supplement: Supplementary file 3 — Supporting Information 3 List of qRT‐PCR primers of genes related to epigenetics in Nile tilapia. [file ANU-2026-8360989-s001.pdf]

Aquaculture Nutrition

Supplementary information

Supporting Information 3: Table S2

The injection of lipids into yolk sac has been shown to alter the lipid metabolism of adult Nile tilapia (*Oreochromis niloticus*)

Linli Luo[a], Sirijanya Thongchaitriwat[a], Suksan Kumkhong[a], Janethida Kiatmontri[a], Shenglin Yang[b], Stephane Panserat[c], Surintorn Boonanuntanasarn[a],*

[a] *School of Animal Technology and Innovation, Institute of Agricultural Technology, Suranaree University of Technology, Nakhon Ratchasima, Thailand 30000*
[b] *Key Laboratory of Animal Genetics, Breeding and Reproduction in the Plateau Mountainous Region, Ministry of Education, Guizhou University, Guiyang, 550025, China*
[c] *National Research Institute for Agriculture Food and Environment, Université de Pau & Pays de L'Adour, NuMeA, Aquapôle, 64310 Saint-Pée-Sur-Nivelle, France.*

* Corresponding author: surinton@sut.ac.th (Surintorn Boonanuntanasarn)

SUPPLEMENTARY TABLE S2: List of qRT-PCR primers of genes related to epigenetics in Nile tilapia

Genes	5'/3' Forward primer	5'/3' Reverse primer	Access numbers
References gene			
ef1α	GCACGCTCTGCTGGCCTTT	GCGCTCAATCTTCCATCCC	AB075952
DNA methylation writers			
dnmt3aaaa	CCAACAACCACGAGCAGGAA	TGCCGACAGTGATGGAGTCT	XM_005475084.4
dnmt3bb	TGCAGGAGTTCTTCGCCAAC	TGCCACATACTGACCCACCT	XM_025901790.1
DNA methylation eraser			
tet1	CATCCAGTCCCAGCACAACC	CTCTATTTGGCGTGCGCTGA	XM_025897345.1
tet2	GCAGCTGCCAACAAGAATGC	TGTTGCTGCTGCTGATGGAC	XM_005457001.3
tet3	GCAAGCCAACCAACCAAACC	GATGTGTTGGCTCCGACCTG	XM_019365521.2
H3K4me3 writer			
setd1a	GGAACTCCGGTCTGGATGGT	CGAAGCTGCCCATCTGTGTT	XM_005468973.4
setd1ba	AAGACAGGGAGGCAGCAGAA	CCTCAGGACTGGGAGGTCTG	XM_005470275.4
kmt2ba	ACTCTGAGGGACCTGGAGGA	AGAGGAGGTGAAGCCGATCC	XM_013275905.3
kmt2bb	GCTCCCGTCAGTGTGTCTTC	TCTGGCTCCAACCCAGTCAA	XM_013277028.3
H3K4me3 eraser			
kdm5bb	CATCCCTGCCTACCTCCCAA	AAGGCTCCAGGTGGACTTGA	XM_003439103.5
kdm5c	CTCTCCACCCTGGAGGCAAT	AGCTACCAGGCCCTCCAAAT	XM_005448517.4
riox1	CCACCTGGCACACAAGGATT	TCCGGCTTCTACCACCACAT	XM_005475002.4
H3K9ac specific writer			
kat2b	GGCCTTTCATGGAGCCTGTG	CTCGCTCTCTGGAGGGTTGT	XM_003444058.3
kat6a	CATCCCGTCCACTGCTTTCC	CCTGTTCACGCTACCACCAC	XM_005472980.3
gtf3c4	CTTGTGGCGGTTCAAGCTCT	GGCTCGCCTTCCTCTTTCAC	XM_003440231.5
H3K9ac specific eraser			
sirt2	GCGAGTCTAGTCAGCAGGGT	CCCAGAAGATCAGCTAGAGCCA	XM_003449264.5
sirt5	ATTTGCCCAGGTGTGAGCAG	GAGCAAACATGGCTGCAGGA	XM_003457306.5
